# A Comprehensive Bioinformatic Analysis for Identification of Myeloid-Associated Differentiation Marker as a Potential Negative Prognostic Biomarker in Non-Small-Cell Lung Cancer

**DOI:** 10.3389/pore.2022.1610504

**Published:** 2022-08-19

**Authors:** Min Zhou, Yan Chen, Xuyu Gu, Cailian Wang

**Affiliations:** Department of Oncology, Zhongda Hospital, School of Medicine, Southeast University, Nanjing, China

**Keywords:** bioinformatics, biomarker, NSCLC, survival-related gene, MYADM

## Abstract

**Objectives:** This study aimed to identify a molecular marker associated with the prognosis of non-small-cell lung cancer (NSCLC).

**Materials and Methods:** The RNA sequencing data and clinical information of NSCLC patients were obtained from The Cancer Genome Atlas (TCGA) and the Gene Expression Omnibus (GEO). The weighted gene co-expression network analysis (WGCNA) was used to identify the co-expression gene modules and differentially expressed genes (DEGs) by comparing gene expression between NSCLC tumor tissues and normal tissues. Subsequently, the functional enrichment analysis of the DEGs was performed. Kaplan-Meier survival analysis and the GEPIA2 online tool were performed to investigate the relationship between the expression of these genes of interest and the survival of NSCLC patients, and to validate one most survival-relevent hub gene, as well as validated the hub gene using independent datasets from the GEO database. Further analysis was carried out to characterize the relationship between the hub gene and tumor immune cell infiltration, tumor mutation burden (TMB), microsatellite instability (MSI), and other known biomarkers of lung cancer. The related genes were screened by analyzing the protein-protein interaction (PPI) network and the survival model was constructed. GEPIA2 was applied in the potential analysis of pan-cancer biomarker of hub gene.

**Results:** 57 hub genes were found to be involved in intercellular connectivity from the 779 identified differentially co-expressed genes. Myeloid-associated differentiation marker (*MYADM*) was strongly associated with overall survival (OS) and disease-free survival (DFS) of NSCLC patients, and high *MYADM* expression was associated with poor prognosis. Thus, *MYADM* was identified as a risk factor. Additionally, *MYADM* was validated as a survival risk factor in NSCLC patients in two independent datasets. Further analysis showed that *MYADM* was nagetively associated with TMB, and was positively correlated with macrophages, neutrophils, and dendritic cells, suggesting its role in regulating tumor immunity. The *MYADM* expression differed across many types of cancer and had the potential to serve as a pan-cancer marker.

**Conclusion:** *MYADM* is an independent prognostic factor for NSCLC patients, which can predict the progression of cancer and play a role in the tumor immune cell infiltration in NSCLC.

## Introduction

Lung cancer is the second most common cancer in humans and the leading cause of cancer-related deaths worldwide. Lung cancer is a heterogeneous disease with many varied pathological types and subtypes which are clinically relevant. Among these, non-small-cell lung cancer (NSCLC) accounts for about 85% of all lung cancer, and lung adenocarcinoma (LUAD) and lung squamous cell carcinoma (LUSC) are the most common pathological subtypes of NSCLC [1].

Traditional treatments for lung cancer include surgery, chemotherapy and radiotherapy, unfortunately, these methods often do not have lasting success. Molecular targeting therapy and checkpoint inhibitor therapy have been important developments in systemic therapy of NSCLC. The identification of pathogenic genes changed the treatment model for lung cancer, which allowed clinicians to conduct individualization of treatment taking account of the genetic backgrounds of the patients. Therefore, deeper and more comprehensive identification of the driver genes of NSCLC and more accurate immunotherapy efficacy prediction is essential to finding better prediction mechanisms and the design of new drugs, which will be beneficial to the prognosis of the patients.

The construction of various cancer databases and the population of bioinformatic methods have made the initial screening of new cancer targets extremely accessible to researchers. In this study, RNA sequencing data from the cancer databases were used to explore the differentially expressed genes (DEGs). By the means of weighted gene co-expression network analysis (WGCNA), functional enrichment analysis, protein-protein interaction (PPI) network analysis and other bioinformatic methods the hub gene was found, as well as its related function, and feasibility as prognostic molecular markers in NSCLC patients.

## Materials and Methods

### Data Acquisition and Processing

The RNA-Seq data and clinical data of NSCLC patients were downloaded from The Cancer Genome Atlas (TCGA) database (https://portal.gdc.cancer.gov/) and NSCLC GSE74706 expression profiling data were downloaded from the Gene Expression Omnibus (GEO) database (https://www.ncbi.nlm.nih.gov/gds) (Table 1). We combined TCGA-LUAD and TCGA-LUSC datasets (henceforth referred to as TCGA dataset) with 108 normal samples and 1037 tumor samples, while GSE74706 included 18 normal samples and 18 tumor samples. The DEGs between TCGA and GSE74706 were identified for further analysis by the “limma” package of the R software (| logFC | > 1, adjusted *p* < 0.05 were used as the criteria). Additionally, the expression profiles and clinical information of GSE50081 (181 Stage I and II NSCLC cases) and GSE8894 (138 cases) datasets were downloaded for subsequent validation of the survival analysis.

**TABLE 1:** Datasets from TCGA database and GEO database.

Dataset ID	Platform	Sample count
TCGA	-	1037(T)/108(N)
GSE74706	GPL13497	18(T)/18(N)
GSE8894	GPL570	138(T)/0(N)
GSE50081	GPL570	181(T)/0(N)

### Identification of Co-Expression Modules by Weighted Gene Co-Expression Network Analysis

In the regulation of biological processes, important functional genes tended to function through co-expression. Co-expression networks facilitate the screening of disease-related gene clusters and can be used to identify therapeutic targets and biomarkers. In this study, WGCNA, a systematic biological method for constructing a scale-free network using gene expression data, was employed. A weighted co-expression network was constructed from the expression profile data of the candidate gene sets by the “WGCNA” package of R. We analyzed the expression profiles of TCGA and GSE74706 to screen for co-expression modules and identify key biomarkers. By using the formula A_IJ_ = | S_IJ_ | β (A_IJ_: adjacency matrix between gene I and Gene J, S_IJ_: similarity matrix obtained by Pearson correlation of all gene pairs, β: soft threshold), and transformed into a topological overlap matrix (TOM) and its related similarity (1-TOM). To divide similar genes into different co-expression modules, a hierarchical clustering tree based on the 1-TOM matrix was constructed.

### The Intersection of Co-Expressed and Differentially Expressed Genes

In the process of screening for functional hub genes, we screened DEGs in TCGA and GSE74706. To find the differentially co-expressed genes, we used the “limma” software package in R with the criteria of | logFC | > 1 and adjusted *p* < 0.05. The “limma” software package enabled us to analyze the microarray data and the differential expression of RNA. The genes in the TCGA and GSE74706 datasets were visualized as volcano plots by “ggplot2”, an R package. Then, the potential biomarkers were identified as belonging to the intersection of DEGs and co-expressed genes from WGCNA, and the results were displayed by the “venn” package of R software.

### Functional Annotation of Differentially Co-Expressed Genes

The “ClusterProfiler” package in R provided the functional annotations and pathway enrichment analysis for the selected genes. Gene Ontology (GO) summarized the three main attributes: biological processes (BP), cell components (CC), and molecular functions (MF), which outlines the biological characteristics of genes. 779 DEGs identified through the differential expression analysis and WGCNA analysis, were functionally annotated to explore the occurrence and development mechanism of the NSCLC.

### The Expression and Validation of Prognostic Value of Hub Genes

The Kaplan-Meier univariate survival analysis was conducted and the R packages “survival” (v3.2.7) and “survminer” (v0.4.8) were used to investigate the relation between hub genes and the overall survival (OS) of patients based on clinical data from the TCGA database. Moreover, the online tool GEPIA2 (http://gepia2.cancer-pku.cn/) was used to analyze the significance of hub genes in the disease-free survival (DFS) of NSCLC patients. In the survival analysis, the survival curve was generated by the Kaplan-Meier method, and the log-rank test was used to determine the statistical significance of the difference.The top 1 gene with the greatest impact on the prognosis was selected as survial-related core gene.

### The Correlation Analysis of Prognosis-Related Gene With Immune Cell Infiltration, Tumor Mutational Burden, Microsatellite Instability and Other Prognosis Markers

The dependence of TMB, MSI, and other prognosis markers on the expression of prognosis-related gene were investigated through the Spearman correlation analysis. Multiple immune cell analysis-related tools such as CIBERSORT, XCELL, EPIC, MCPCOUNTER, and QUANTISEQ were used to analyze the correlation between the expression profile of hub gene and immune cell infiltration. The correlation of prognosis-related gene with B cells, CD4 + T cells, CD8 + T cells, neutrophils, macrophages, dendritic cells were analyzed by the TIMER database (https://cistrome.shinyapps.io/timer/), and the correlation curves were downloaded.

### Validation by Independent Datasets

Two independent datasets from GEO were used to validate by survival analysis and to verify that the hub gene derived from the process described thus far was still valid for other datasets. We downloaded the expression data and the clinical data of GSE50081 and GSE8894 datasets from the GEO for survival analysis to verify the validity of the previously obtained results.

### Protein-Protein Interaction Network Construction

The R package, “STRINGdb” based on the STRING database (https://STRING-db.org/) the protein-protein interactions were identified, a PPI network of the hub was constructed, and visualized as a network diagram.

### Construction of the Survival Model

The survival model was built using hub gene modules obtained from the STRINGdb, and the results were visualized using the R package “survival,” “survminer,” and “ggrisk.”

### Analysis of the Key Gene to Evaluate the Potential as Pan-Cancer Biomarkers

The online tool GEPIA2 was employed to analyze the expression differences of the selected hub gene between the cancer samples and the normal samples to determine whether it had the potential to act as pan-cancer biomarker.

## Results

### Construction of Co-Expression Module in Weighted Gene Co-Expression Network Analysis

**FIGURE 1:**
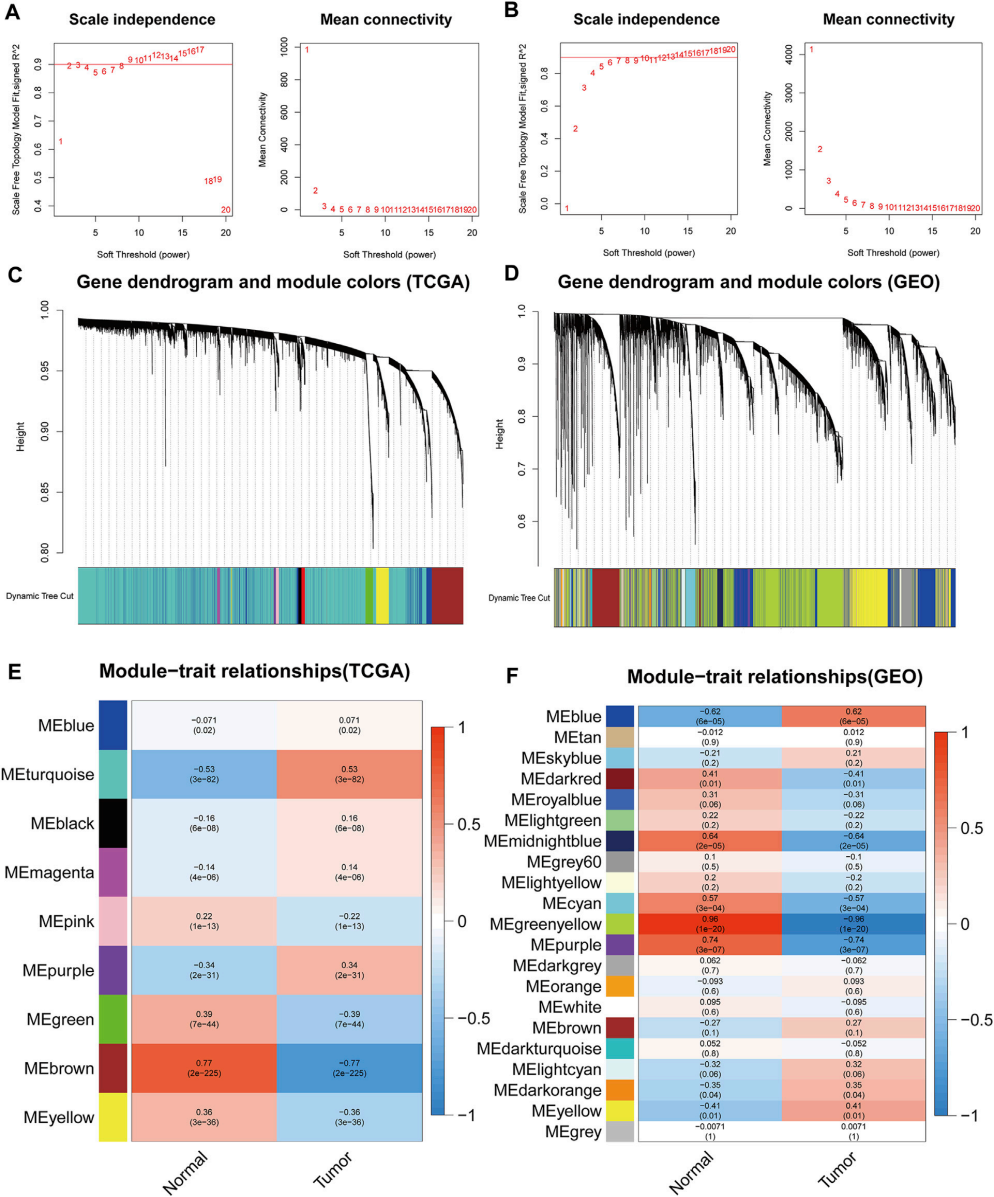
The weighted gene co-expression network analysis (WGCNA) of TCGA and GSE74706 datasets. **(A,B)** The soft threshold selection diagram for network topology analysisfrom the TCGA/GSE74706 datasets; **(C,D)** Construction of the network and the module detection diagramfrom the TCGA/GSE74706 datasets; **(E,F)** The correlation between the modules and the disease/normal groupfrom the TCGA/GSE74706 datasets.

To recognize highly significant co-expression module genes, we correlated them with normal and tumor groups, and selected a soft threshold of β = 2 and 6 by the function pickSoftThreshold to establish a scale-free network.In this study, night modules of TCGA and 20 modules of GSE74706 were identified (excluding grey module which contained genes were not assigned to any functional group). In addition, we assessed the correlation between each module and differences between the normal and tumor groups by plotting the correlation heat maps. The results showed that the most discernable difference was between the brown module in TCGA and the greenyellow module in GSE74706. As a result, these highly relevant modules were considered candidates associated with the clinical characterization and were used in the subsequent analysis (Figure 1).

### Identification of Differentially Co-Expressed Genes

**FIGURE 2:**
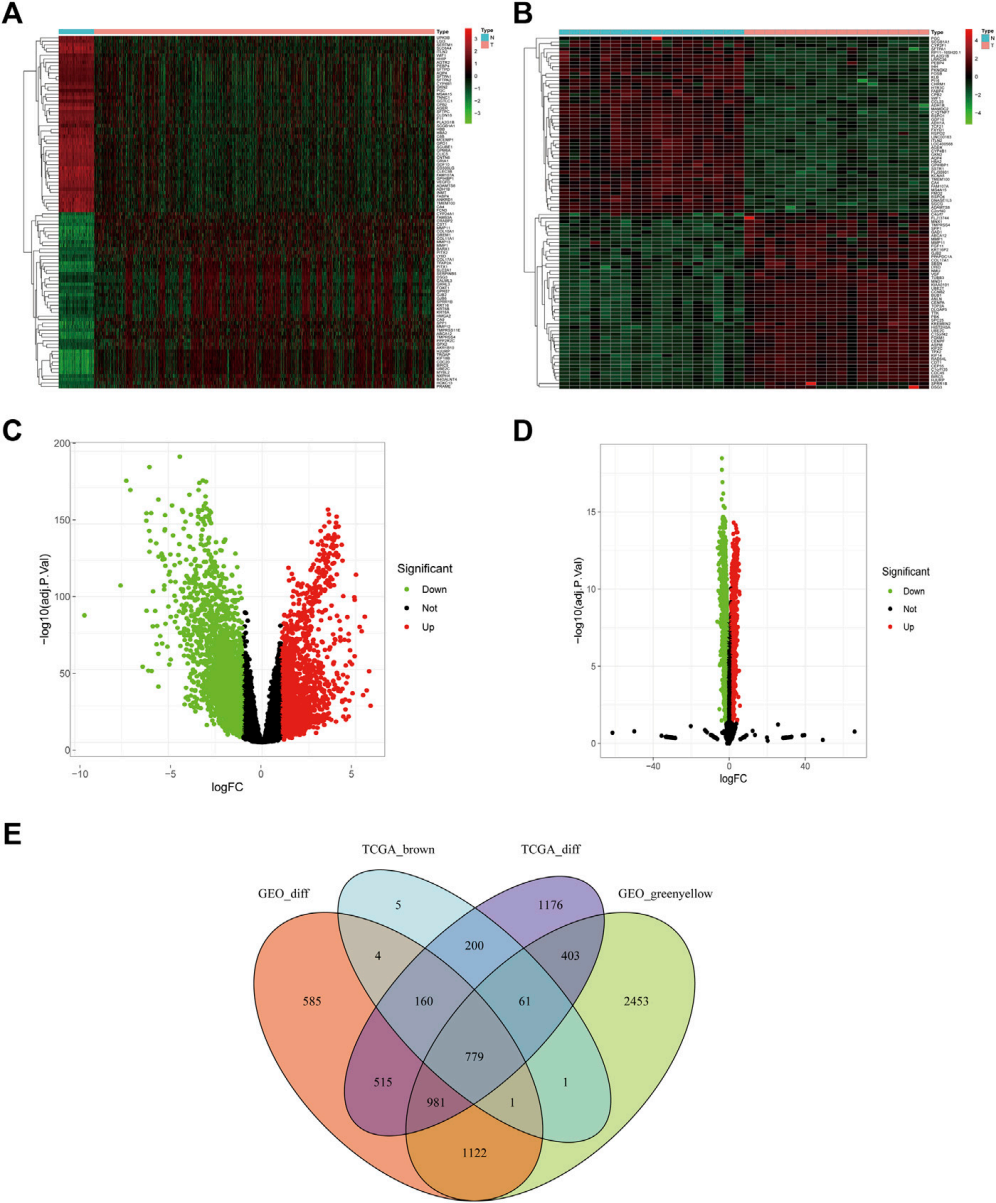
Differentially co-expressed genes of TCGA and GSE74706 datasets. **(A)** The heat map of the differential genes screened from the TCGA dataset; **(B)** The heat map of the differential genes screened from the GSE74706 dataset; **(C,D)** The volcano plot based on the Fold-Change and *p*-value of the genes from the TCGA/GSE74706 datasets; **(E)** Venn diagram of the intersection based on the analysis of multiple data sets.

With | logFC | > 1, adjusted *p* < 0.05 as the determining criteria, 4,275 DEGs were identified from the TCGA dataset, and 4,147 DEGs were identified from the GSE74706 dataset. The brown and greenyellow modules comprised 1,211 and 5,801 genes, respectively. A total of 779 differentially co-expressed genes (Figure 2) were obtained from the intersection of the above four groups.

### Functional Enrichment Analysis of Differentially Co-Expressed Genes

**FIGURE 3:**
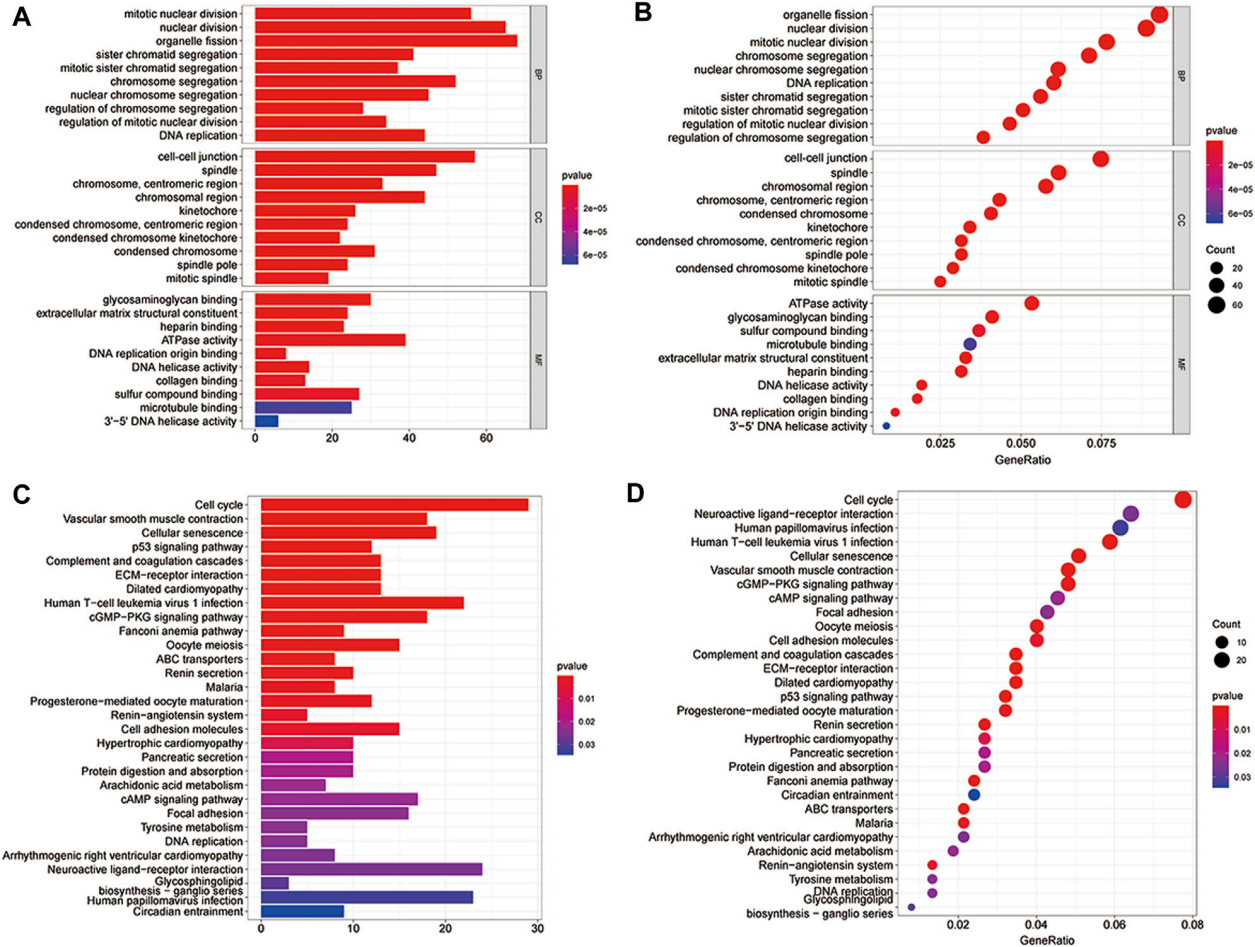
Gene ontology (GO) and Kyoto Encyclopeia of Genes and Genomes (KEGG) pathway enrichment of the differentially co-expressed genes. **(A,B)** The bar and bubble plots of the GO enrichment; **(C,D)** The bar and bubble plots of KEGG enrichment.

To further understand the biological function of the 779 differentially co-expressed genes involved, the R “ClusterProfiler” package was used for functional enrichment analysis. Previous experiments have shown that cell-cell junction functions connected cells in tissues and regulate tissue barrier function, cell proliferation, and migration. Defects in cell-cell junctions cause widespread tissue abnormalities and disrupt the balance, which was common in genetic abnormalities and cancers [2]. Accordingly, we took the genes enriched in the functional pathways of cell-cell junctions as important gene sets for subsequent analysis. The results of functional enrichment showed that a total of 57 genes were enriched in the functional pathways of the cell-cell junctions (Figure 3).

### Validation of Intercellular Hub Gene Expression Patterns and the Prognostic Value

**FIGURE 4:**
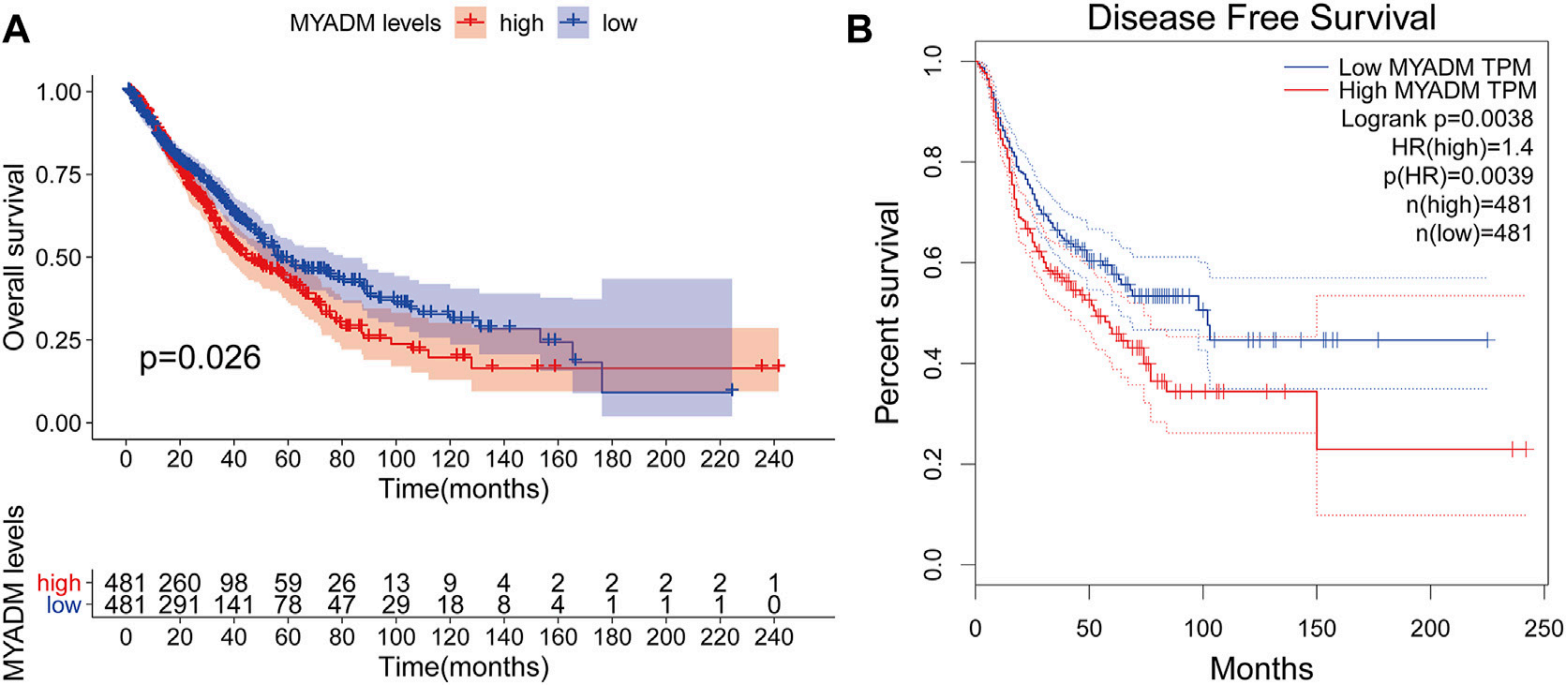
The prognostic survival curve for the high-risk group and low-risk group. **(A)** Kaplan-Meier survival curve based on OS; **(B)** Kaplan-Meier survival curve based on DFS.

Univariate Cox regression analysis was used to screen out the genes with a significant relationship with survival. The results showed that myeloid-associated differentiation marker (*MYADM*) was the most correlated gene with the prognosis of the patients (*p* = 0.02617). Repeated validation through the OS and DFS survival analyses indicated *MYADM* as a risk factor among the 57 hub genes and was negatively correlated with survival (Figure 4).

### The Correlation of Immune Cell Infiltration, Tumor Mutation Burden and Microsatellite Instability to the Survival-Related Gene

**FIGURE 5:**
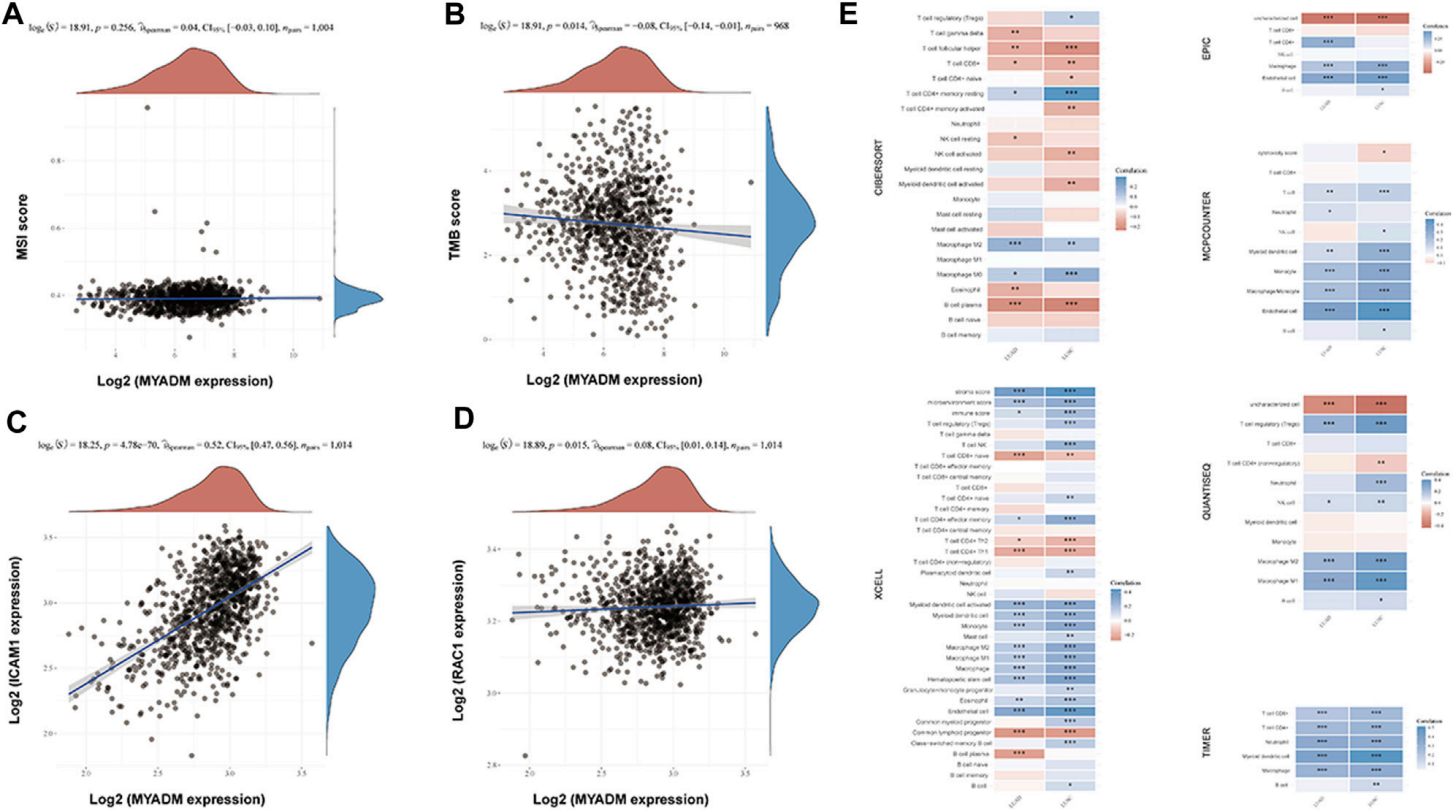
Correlation analysis of the expression of the *MYADM* with immune cell infiltration levels, TMB, MSI, and other prognostic markers in NSCLC. **(A,B)** The scatters plot showing the correlation between *MYADM* and MSI, TMB; **(C,D)** The scatter plots showing the correlation between *MYADM* and biomarkers *ICAM1* and *RAC1* in NSCLC; **(E)** The heat map of correlation between *MYADM* and different immune cells in multiple immune data sets.

The Spearman correlation analysis was performed to study the relationship between the *MYADM* expression and immune cell infiltration, TMB, and MSI. The results showed that *MYADM* was weak negatively correlated with TMB (*p* = 0.014, *r* = −0.08), but not with MSI (*p* = 0.256, *r* = 0.04), TMB has been found in a variety of tumor immunotherapy in recent years as an independent biomarker that can be used to predict the efficacy of immunotherapy. MYADM has a weak negative correlation with TMB levels, suggesting that MYADM may play a role in predicting the efficacy of immunotherapy (Figures 5A,B). Additionally, we obtained the NSCLC biomarkers from previous studies [3, 4] and analyzed their correlation with *MYADM*. It was found that *MYADM* had a high correlation with intercellular adhesion molecule-1 (*ICAM1*) and a weak correlation with RAS-related C3 botulinum toxin substrate 1 (*RAC1*), which demonstrated the potential of *MYADM* as a biomarker for NSCLC (Figures 5C,D). Furthermore, we analyzed the relationship between the risk gene *MYADM* and various types of immune cells in multiple immune datasets. It showed that *MYADM* was a significant correlation with immune cells in multiple immune datasets. In both LUSC and LUAD, *MYADM* was positively associated with macrophage M2 within the CIBERSORT and negative with T cell follicular helper and B cell plasma. In the XCELL, *MYADM* was positively correlated with myeloid dendritic cell activated, myeloid dendritic cell, monocyte, macrophage M2, macrophage M1, hematopoietic stem cell, eosinophil immune cells, while it was negatively correlated with T cell CD8 + naive, T cell CD4 + Th1, and common lymphoid progenitor. *MYADM* was positively correlated with immune cells (macrophage) in EPIC, immune cells (T cell, myeloid dendritic cell, monocyte, macrophage, monocyte) in MCPCOUNTER, immune cells (macrophage) in QUANTISEQ, immune cells (T cell CD8 +, T cell CD4 +, neutrophil, myeloid dendritic cell, and macrophage) in TIMER (Figure 5E).

**FIGURE 6:**
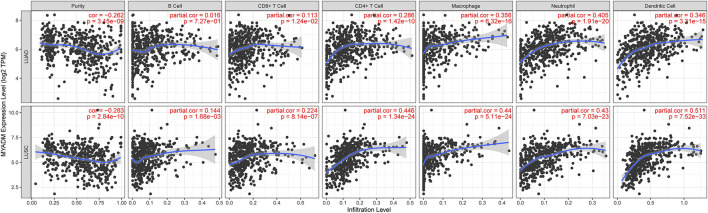
Correlation of *MYADM* with tumor purity, B cells, CD4 + T cells, CD8 + T cells, neutrophils, macrophages and dendritic cells by TIMER database in lung adenocarcinoma (LUAD) and lung squamous cell carcinoma (LUSC) patients.

To further study the relationship between *MYADM* and the immune microenvironment of lung cancer, we analyzed the relationship between *MYADM* and B cells, CD4 + T cells, CD8 + T cells, neutrophils, macrophages, dendritic cells, and tumor purity, and obtained the correlation coefficient by the TIMER online resource. The scatter plot showed that the *MYADM* was associated with macrophages, neutrophils, and dendritic cells in both LUSC and LUAD (Cor >0.3, *p* < 0.05) (Figure 6).

### Survival Analysis in Independent Datasets

**FIGURE 7:**
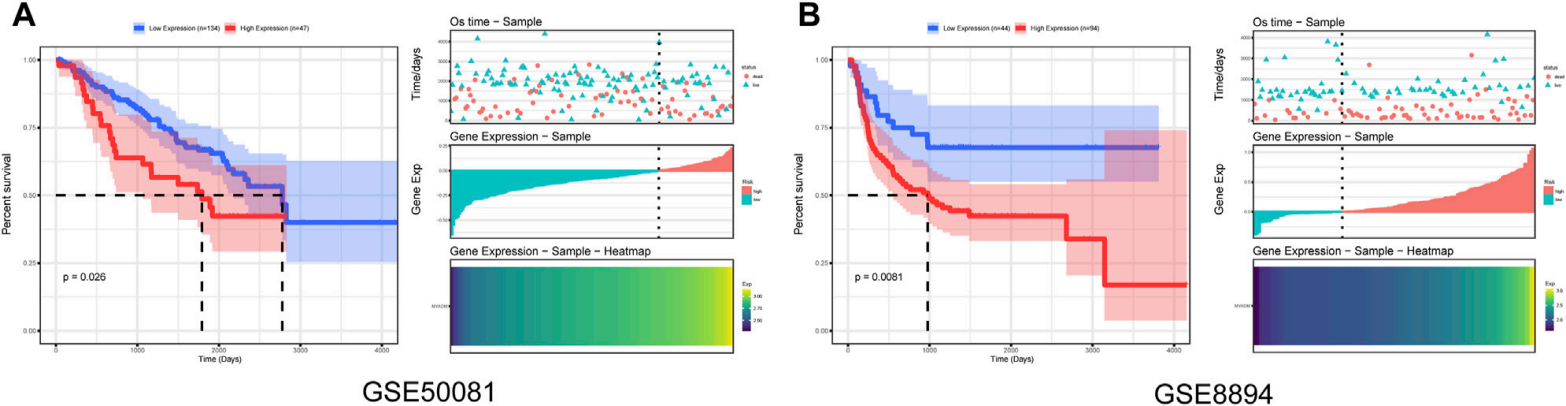
Prognostic survival curves in the *MYADM* high-expression group and low-expression group. **(A)** Kaplan-Meier curve based on GSE50081; **(B)** Kaplan-Meier curve based on GSE8894.

Furthermore, to evaluate the reliability of *MYADM* as a risk factor, we downloaded the gene expression data and the clinical data of GSE50081 and GSE8894 in the GEO database and performed the univariate Cox regression analysis of the hub gene *MYADM*. In this study, the minimum *p*-value method, which has been proven to be effective in many fields [5], was used to obtain the best grouping values and to generate the Kaplan-Meier survival curves. The results showed that *MYADM* was a risk factor according to two both of these datasets and negatively impacted the prognosis of patients, further indicating that *MYADM* could be used as a biomarker for NSCLC (Figure 7).

### Construction of the Survival Model

**FIGURE 8:**
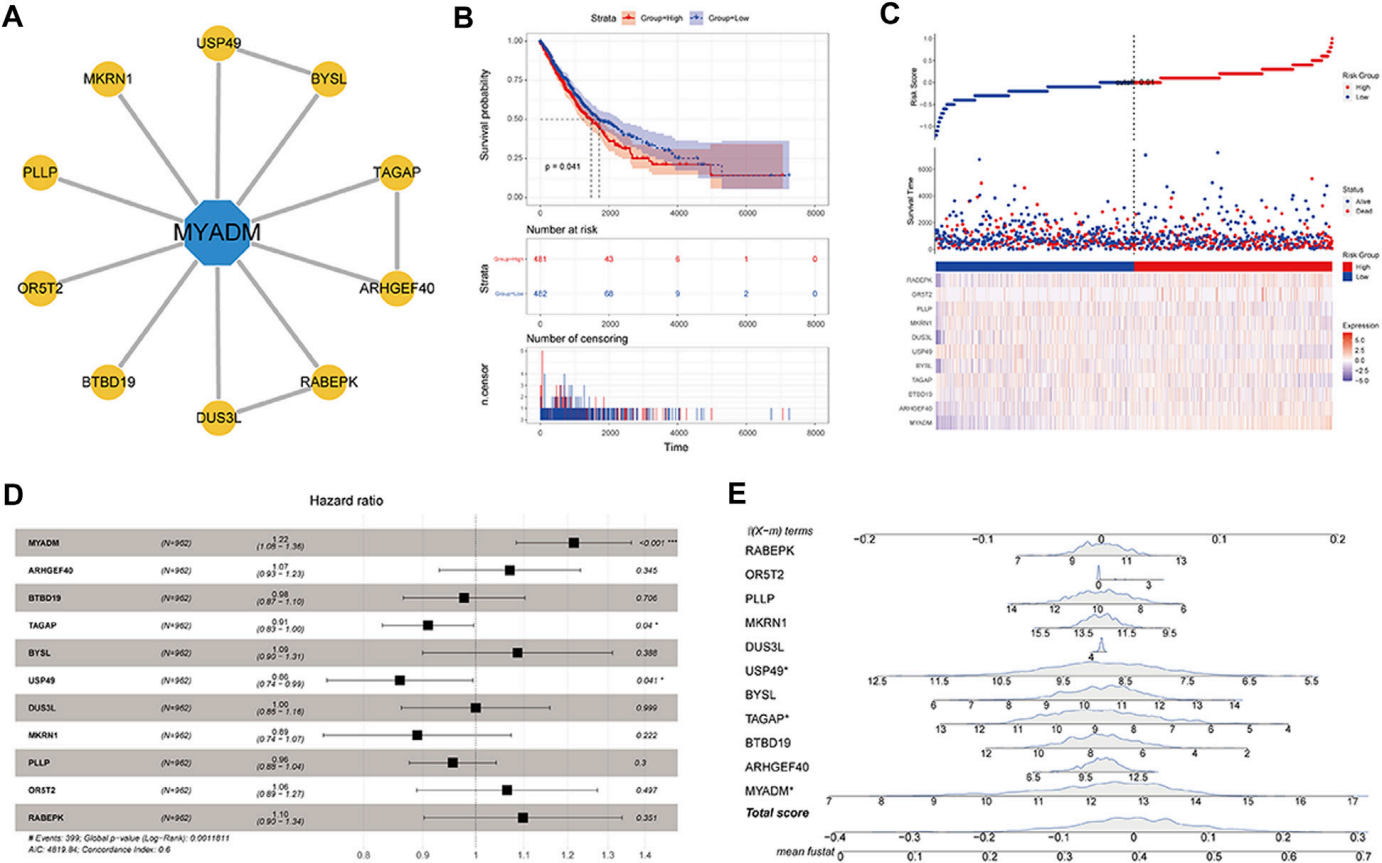
Construction of the *MYADM* gene-related survival model. **(A)** *MYADM* interactive gene network map; **(B)** Kaplan-Meier survival curve based on candidate gene set risk model; **(C)** The risk score map based on integrated gene set; **(D,E)** The forest plot and nomogram based on integrated gene set.

After determining that *MYADM* would negatively impact the patient’s prognosis, STRINGdb was used to predict the PPI relationship of the *MYADM* gene (Figure 8A), establish the survival model for the candidate genes, and apply the risk score model:

$$Risk\;score=\overset{n}{\underset{i=1}{\sum }}\beta_{i}Exp\left(C_{i}\right)$$

Among them, β_i_ was the Cox regression coefficient of each RNA (expressed as Ci), n was the number of RNA in the gene set, Exp (C_i_) was the RNA C_i_ expression value in the corresponding sample. Then by calculating the sample risk score by the above formula, the patients were divided into the high-risk group and the low-risk group by taking the median as the node. By the result of the survival curve presented in Figure 8B, the survival difference of the high-risk and low-risk groups was significant, and risk model genes can be used as biomarkers to predict the prognosis of patients. We also plotted the risk factor graph, forest plot, and nomogram showing the results (Figures 8C–E). In Figures 8D,E, it can be seen that *MYADM*, T cell activation rhoGTPase activating protein (*TAGAP*), and ubiquitin specific peptidase 49 (*USP49*) had relatively greater weights in the model, where *MYADM* was the risk factor, suggesting that it had a greater influence on the prognosis of patients.

### Pan-Cancer Analysis

**FIGURE 9:**
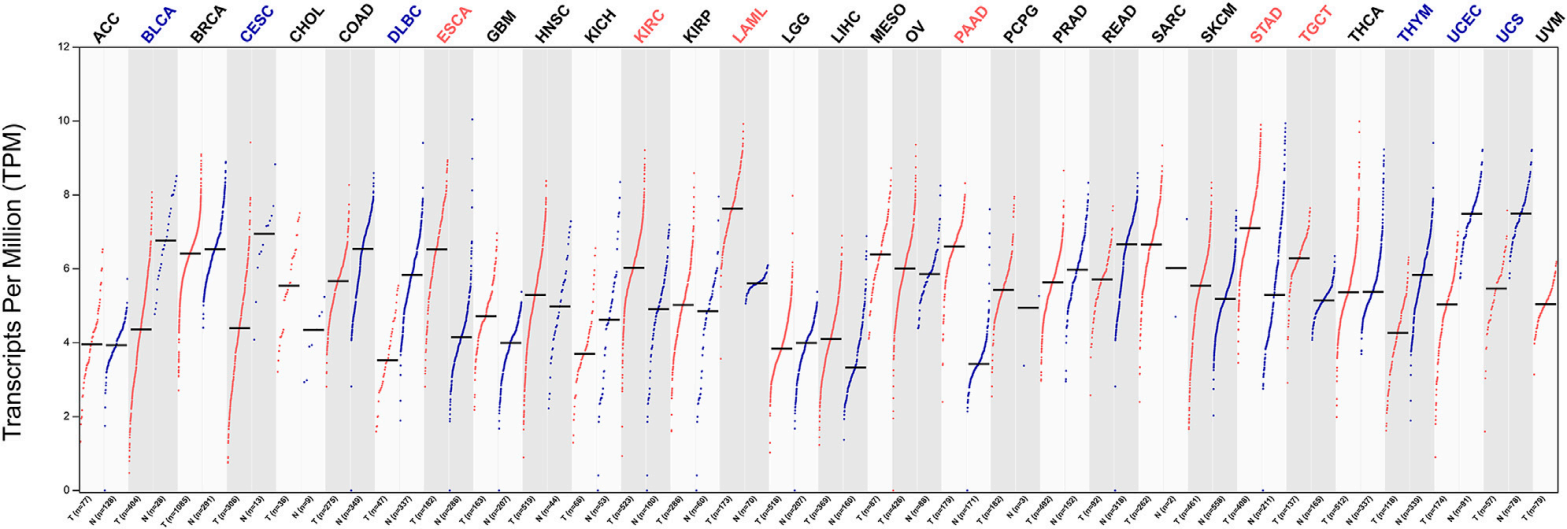
Gene expression in normal/patients of various types of cancer.

After identifying *MYADM* as a potential biomarker for NSCLC, we analyzed the expression of *MYADM* in 31 other tumors, among these *MYADM*, was highly expressed in 6 tumors, including esophageal carcinoma, kidney renal clear cell carcinoma, acute myeloid leukemia pancreatic adenocarcinoma, stomach adenocarcinoma and testicular germ cell tumors, and lowly expressed in 6 tumors such as bladder urothelial carcinoma, cervical squamous cell carcinoma and endocervical adenocarcinoma, lymphoid neoplasm diffuse large B-cell lymphoma, thymoma, uterine corpus endometrial carcinoma, uterine carcinosarcoma. It has been shown that the expression of *MYADM* was different across various types of cancer, which indicates that *MYADM* has the potential of being a pan-cancer biomarker (Figure 9).

## Discussion

At present, lung cancer is an important cause of malignant tumor death in the world, and its diagnosis rate and mortality rate remain high. Despite the improvement in the treatment of NSCLC in recent years, the prognosis of patients is still not ideal. It is of potentially clinical significance to understand the molecular mechanism of tumor progression, which can predict the prognosis of NSCLC patients accurately, and enable making individual treatment plans based on their genetic profile. This would be potentially beneficial to the prognosis of patients suffering from NSCLC.

During the discovery phase of our study, we selected datasets of NSCLC tissue and normal lung tissue from the TCGA and GEO databases. There were 779 differentially co-expressed genes screened from the two datasets, and the enrichment analysis results showed that the differentially co-expressed genes were enriched in organelle fission, mitosis, nuclear division, and chromosome segregation in the BP region, the intercellular junction, spindle, chromosome region in the CC region and ATPase activity in MF region.

Cell-cell junction is an important site for the interaction and synergy between adjacent cells in a multicellular organism through the cell plasma membrane, which connects similar cells into tissues and is kept relatively stable between adjacent tissues cells. The abnormal cell-cell junctions may play an important role in tumorigenesis. In our present study, 57 core genes were enriched in the cell-cell junction gene expression module, and further survival analysis revealed that the gene most related to prognosis was *MYADM*.

*MYADM* belongs to the MAL family and maps to the human chromosome 19q 13.33-q 13.4. It consists of 3 exons and 2 introns, and it spans a 7.1-Kb genomic region [6]. The MYADM protein is located in the nuclear envelope and cytoplasmic inner membrane, forming a complete membrane protein [7, 8]. Previous studies have found that *MYADM* is selectively expressed in myeloid cells [9], participates in the process of myeloid differentiation [6], and relates to the differentiation of hematopoietic cells. Moreover, *MYADM* is the target gene of c-Myb which is an important regulator of the hematopoietic cell development [10]. Additionally, it may play a role in cell migration through the development of lamellipodium [8].

Aranda JF et al. detected the expression of *MYADM* in several tumor cell lines [8]. The expression of MYADM protein was up-regulated in metastatic melanoma and hepatocellular carcinoma tissues [11, 12]. Cancer is driven by genetic change, and the wealth of data to systematically record this variation on a genome-wide scale provides an important opportunity to develop a comprehensive picture of commonalities, differences, and emerging themes across cancer lineages. After pan-cancer analysis, it was found that the expression levels of MYADM in different cancer types were different, some of which were up-regulated and others were down-regulated, indicating that MYADM plays multiple roles in pan-cancer. Moreover, *MYADM* was essential for tumor cell proliferation and migration [8]. Papasotiriou I et al. found that the expression of *MYADM* is up-regulated in differentially expressed genes when hormone-refractory prostate cancer cells are co-cultured with osteoblasts or endothelial cells, suggesting that it is related to the process required for metastasis [13]. The expression level of *MYADM* mRNA is significantly increased during the differentiation of myeloid leukemia, and thus, can be used as a membrane marker for disease surveillance [9]. The *MYADM* gene was found to be associated with 5 years of biochemical recurrence in prostate cancer in most African American biomarkers studies lacking E26 transforming specific family fusion events [14].

*MYADM* has not been further studied in NSCLC. Our findings suggested that *MYADM* may be a potential target for the study of pathogenesis, diagnosis, treatment, and prognosis of NSCLC. This study provided an important theoretical basis for the further study of NSCLC *in vivo* and *in vitro* experiments and the search of molecular markers for clinical diagnosis and treatment of NSCLC.

Currently, immunotherapy has been the focus of NSCLC treatment, predictive cancer biomarkers are important for assessing benefit in relation to individual patients, accurately stratifying the population most likely to benefit from targeted therapy. At present, PD-L1 expression level, MSI, high tumor mutational burden (TMB-H) may be related to the effect. Our results showed that there was a weak negative correlation between *MYADM* and TMB in NSCLC, but there was no significant correlation with MSI and CD8 + T cells. It was found that immunotherapy in the treatment of lung adenocarcinoma patients with TMB-H resulted in a significantly better prognosis. But in metastatic lung squamous cell carcinoma, immunotherapy prognosis with low tumor mutation load (TMB-L) was even better than that with TMB-H [15]. The correlation may require further discussion and subdivision of pathologic types, and whether *MYADM* can be used as a predictor of immunotherapeutic efficacy remains to be tested.

It is known that the increase of ICAM-1 expression may indicate the poor prognosis in lung cancer patients, which played an important role in the development and metastasis of lung cancer [16]. Previously, Rac1 has been shown to have high expression in different types of tumors, which was associated with poor prognosis, and its high expression in NSCLC stem cells enhanced the malignant behavior of tumor cells [4, 17]. Aranda JF et al found that the decline in the barrier function of *MYADM* silenced cells depended on the expression of ICAM-1 [18], and co-localization of the *MYADM* and the Rac1 to participate in cell migration through the membrane [8]. In our study, comparing *MYADM* with other known lung cancer biomarkers by bioinformatic methods, we found *MYADM* was associated with *ICAM1* and *RAC1*, suggesting that *MYADM* may be a prognostic factor for NSCLC. Additional GEO NSCLC datasets confirmed that *MYADM* acted as an independent risk factor for the survival and prognosis of NSCLC.

In the further analysis of *MYADM*, the survival model was established by searching for interaction genes through the PPI network. The prognosis of the high-risk score group was significantly worse than that of the low-risk score group, in which MYADM, USP49, and TAGAP had the important weights, the former was the risk factor, while the latter two were the protective factors. At present, the research on USP49 is limited and its function in malignant tumors is not completely clear. Luo and Tu et al. showed that USP49 can inhibit the development of pancreatic cancer and could be the tumor suppressor of colon cancer [19, 20]. In suspension Chinese hamster ovary cells, TAGAP acts as a mediator of intracellular cytoskeleton signal to cell surface integrin, and the increase of TAGAP expression enhanced cell proliferation, viability, and adaptability to suspension [21, 22]. The direct or indirect interaction between MYADM and USP49 or TAGAP warrants further discussion.

This study identified one key gene, *MYADM*, as the most relevant to the prognosis for the cellular component in NSCLC through the means of bioinformatic analysis. However, the above results still lack further confirmation of laboratory molecular biology experiments, which is a limitation of the study. Therefore, the subsequent research should focus on the expression and related functions of *MYADM* in NSCLC to provide an adequate understanding of the occurrence and development mechanism and the treatment targets of NSCLC.

## Conclusion

In summary, this study conducted a bioinformatic analysis of RNA sequencing data and clinical data from NSCLC and found that high expression of *MYADM* was associated with poor prognosis in NSCLC. These findings further enhanced the understanding of NSCLC prognosis and may promote risk-stratified disease management. In the future, *MYADM* may be a potential prognostic marker for lung cancer.

## Data Availability

The original contributions presented in the study are included in the article/supplementary material, further inquiries can be directed to the corresponding author.
